# The global regulation of c‐di‐GMP and cAMP in bacteria

**DOI:** 10.1002/mlf2.12104

**Published:** 2024-03-11

**Authors:** Cong Liu, Rui Shi, Marcus S. Jensen, Jingrong Zhu, Jiawen Liu, Xiaobo Liu, Di Sun, Weijie Liu

**Affiliations:** ^1^ Jiangsu Key Laboratory of Phylogenomics & Comparative Genomics, School of Life Sciences Jiangsu Normal University Xuzhou China; ^2^ Key Laboratory of Metabolic Engineering and Biosynthesis Technology, Ministry of Industry and Information Technology Nanjing University of Science and Technology Nanjing China

**Keywords:** bacteria, cAMP, c‐di‐GMP, cross‐regulation, CRP

## Abstract

Nucleotide second messengers are highly versatile signaling molecules that regulate a variety of key biological processes in bacteria. The best‐studied examples are cyclic AMP (cAMP) and bis‐(3′–5′)‐cyclic dimeric guanosine monophosphate (c‐di‐GMP), which both act as global regulators. Global regulatory frameworks of c‐di‐GMP and cAMP in bacteria show several parallels but also significant variances. In this review, we illustrate the global regulatory models of the two nucleotide second messengers, compare the different regulatory frameworks between c‐di‐GMP and cAMP, and discuss the mechanisms and physiological significance of cross‐regulation between c‐di‐GMP and cAMP. c‐di‐GMP responds to numerous signals dependent on a great number of metabolic enzymes, and it regulates various signal transduction pathways through its huge number of effectors with varying activities. In contrast, due to the limited quantity, the cAMP metabolic enzymes and its major effector are regulated at different levels by diverse signals. cAMP performs its global regulatory function primarily by controlling the transcription of a large number of genes via cAMP receptor protein (CRP) in most bacteria. This review can help us understand how bacteria use the two typical nucleotide second messengers to effectively coordinate and integrate various physiological processes, providing theoretical guidelines for future research.

## INTRODUCTION

Nucleotide second messengers are critical regulatory molecules ubiquitous in living organisms, including plants, animals, and microorganisms. Numerous nucleotide second messengers have been discovered in bacteria, which regulate diverse biological processes in response to intracellular and extracellular cues, allowing bacteria to adapt to a wide range of living environments^1^, ^2^, ^3^, ^4^, ^5^. Bis‐(3′–5′)‐cyclic dimeric guanosine monophosphate (c‐di‐GMP) and cyclic AMP (cAMP) are well‐established global regulators, both of which are ubiquitous and immensely versatile signaling molecules that regulate bacterial biological functions at multiple levels and are highly integrated with other global regulatory systems^2^, ^6^. Although numerous reviews summarize the c‐di‐GMP and cAMP regulatory models, reviews that systematically compare global regulatory frameworks of the two nucleotide second messengers are limited. In this review, we show the differences in the global regulatory framework for c‐di‐GMP and cAMP in bacteria. Different signals control intracellular c‐di‐GMP levels via a large number of c‐di‐GMP metabolic enzymes, and c‐di‐GMP exerts its global regulation through a huge number of effectors with varying activities. As these c‐di‐GMP effectors are the essential proteins involved in various physiological processes, the c‐di‐GMP signal controls a range of biological processes at the level of transcription, translation, and protein–protein interaction. cAMP metabolic enzymes are controlled at multiple levels, allowing intracellular cAMP levels to respond to more signals. The cAMP receptor protein (CRP) is also controlled at various levels and exerts its global regulation primarily via controlling the transcription of numerous genes.

## THE GLOBAL REGULATION OF c‐di‐GMP IN BACTERIA

In bacteria, c‐di‐GMP is an intracellular nucleotide second messenger that controls a variety of cellular functions, including biofilm formation, cell cycle, and virulence^7^, ^8^, ^9^, ^10^, ^11^, ^12^. c‐di‐GMP is a “switch molecule” that regulates the “lifestyle transition” from motility to biofilm^9^. Low intracellular c‐di‐GMP levels lead to a planktonic lifestyle, whereas high levels promote biofilm formation^13^. c‐di‐GMP is produced by diguanylate cyclases (DGCs) with a conserved GGDEF domain and degraded by phosphodiesterases (PDEs) with an EAL or HD‐GYP domain^5^. Most bacteria possess multiple DGCs/PDEs, which control both global and local c‐di‐GMP signaling, allowing them to respond to a variety of internal or environmental cues^9^, ^14^, ^15^, ^16^. Several DGCs/PDEs contribute to the global c‐di‐GMP pool by altering total intracellular c‐di‐GMP levels^9^. In local c‐di‐GMP signaling, knocking out a particular DGC or PDE drastically alters a specific phenotype even while intracellular c‐di‐GMP levels are unaffected^9^, ^17^. c‐di‐GMP can perform a regulatory function by acting as a riboswitch ligand^18^, ^19^, ^20^. For instance, c‐di‐GMP regulates the expression of genes involved in swimming and surface motility by binding to multiple riboswitches in *Clostridioides difficile* ^21^, ^22^. Alternatively, c‐di‐GMP can regulate physiological processes by interacting with protein effectors^23^. Some proteins containing the PilZ domain or degenerate GGDEF or EAL domains have been identified as c‐di‐GMP effectors, but most c‐di‐GMP effectors have nonconserved binding sites and motifs^24^, ^25^, ^26^, ^27^. c‐di‐GMP effectors respond to not only global intracellular c‐di‐GMP levels but also local c‐di‐GMP signaling^27^. By binding c‐di‐GMP to these effectors, the intracellular c‐di‐GMP signal controls a range of biological processes at transcription, translation, and protein–protein interaction levels^27^, ^28^, ^29^, ^30^.

Some key transcription factors serve as c‐di‐GMP effectors to influence target gene expression in response to intracellular c‐di‐GMP levels^28^. In *Pseudomonas aeruginosa*, the transcription factor FleQ (flagellar export regulator) is a c‐di‐GMP effector that controls the transcription of genes involved in motility and biofilm formation^8^, ^29^. At low intracellular c‐di‐GMP levels, FleQ functions as a master activator to trigger flagellar gene expression while repressing the transcription of the *pel*, *psl*, and *cdr* genes that are responsible for producing exopolysaccharides and adhesins^8^. At high intracellular c‐di‐GMP levels, the FleQ‐c‐di‐GMP complex promotes the expression of genes involved in biofilm formation while downregulating the expression of the majority of FleQ‐regulated flagellar genes^8^. Consequently, FleQ regulates the lifestyle transition of *P. aeruginosa* between planktonic growth and biofilm formation in response to intracellular c‐di‐GMP levels^8^. Despite many transcription factors having been identified as c‐di‐GMP effectors, their regulatory models in response to c‐di‐GMP are different. In *Vibrio cholerae*, the transcription factors VpsT and VpsR are both c‐di‐GMP effectors, both of which directly regulate the transcription of genes necessary for *Vibrio* polysaccharide synthesis^31^. Direct interaction with c‐di‐GMP is required for VpsT dimerization and DNA binding^32^, ^33^. However, the way that c‐di‐GMP regulates VpsR is quite different. c‐di‐GMP does not significantly improve the affinity between VpsR and DNA^34^. Instead, the correct DNA/VpsR/RNA‐polymerase complex necessary to initiate transcription must be generated by c‐di‐GMP binding to VpsR^34^, ^35^. In addition to its role as a transcription factor, the c‐di‐GMP effector can control gene transcription in diverse ways. *Streptomyces* has a complicated life cycle and development, which is regulated by intracellular c‐di‐GMP levels^36^, ^37^, ^38^. Low intracellular c‐di‐GMP levels speed up sporulation, whereas high levels lock the bacteria into the vegetative development stage^36^, ^37^, ^38^. In *Streptomyces venezuelae*, the c‐di‐GMP effector RsiG (regulator of sigma WhiG) acts as an anti‐σ factor to regulate the expression of sporulation‐related genes^39^. σ^WhiG^, a sporulation‐specific σ factor that indirectly activates the transcription of more than 100 late sporulation genes, controls the differentiation of reproductive aerial hyphae into spores^39^, ^40^, ^41^. Anti‐σ factor RsiG regulates the transcriptional regulatory activity of σ^WhiG^, whereas c‐di‐GMP controls the interaction between RsiG and σ^WhiG^ ^39^. When intracellular c‐di‐GMP levels are high, RsiG and c‐di‐GMP combine to form a complex that interacts with σ^WhiG^ to delay sporulation^39^. RsiG is unable to bind to σ^WhiG^ in the absence of c‐di‐GMP, and σ^WhiG^ is thus released for the activation of late sporulation regulators^39^. The use of transcription factors or transcription‐related proteins as c‐di‐GMP effectors has the advantage of simultaneously activating genes involved in multiple signal transduction pathways or metabolic pathways in response to a single c‐di‐GMP signal, thereby contributing to the transition of the bacterial lifestyle.

**Figure 1:**
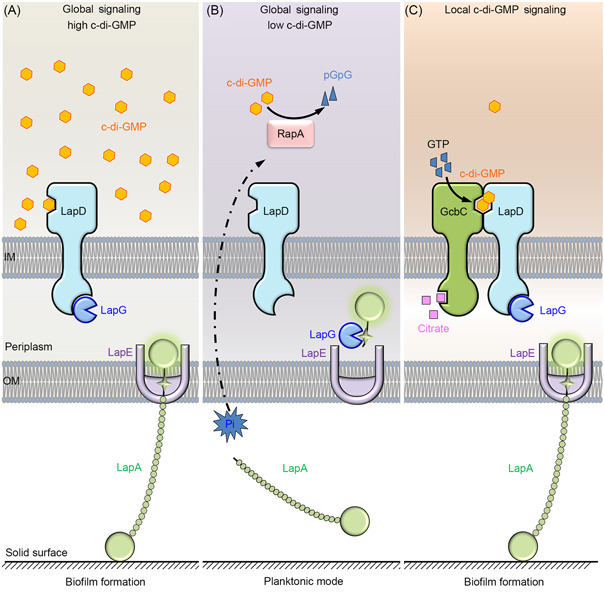
The regulatory model of Lap system in *Pseudomonas fluorescens*. (A, B) Lap system regulates bacterial lifestyle transitions between biofilm and motility in response to global c‐di‐GMP signaling. (C) Local c‐di‐GMP signaling promotes biofilm formation by regulating the Lap system. Dashed arrow indicates indirect regulation. IM, inner membrane; Lap, large adhesion protein; OM, outer membrane; Pi, inorganic phosphate.

In addition to regulating transcription, the activity of c‐di‐GMP effectors changes allosterically upon c‐di‐GMP binding and thus regulates a defined interacting target protein. The Lap (large adhesion protein, LapAGD) system, which has been extensively studied in *Pseudomonas fluorescens*, controls the bacterial lifestyle transition between biofilm and motility in response to internal c‐di‐GMP levels (Figure 1)^27^. LapA is a cell‐surface localization adhesive protein that regulates surface adherence and aids in biofilm stabilization^42^. LapA is secreted to the cell surface by the type I secretion system (T1SS)^27^, ^42^. In addition, LapA is anchored to the cell surface via a TolC‐like outer membrane pore LapE^27^. LapG is a periplasmic protease that can cleave the N‐terminus of LapA, liberating the cell from the surface^43^, ^44^, ^45^. LapD is an inner‐membrane‐spanning c‐di‐GMP effector with two degenerate c‐di‐GMP‐metabolizing domains: a GGDEF domain and an EAL domain^46^, ^47^, ^48^. When the cytoplasmic concentration of c‐di‐GMP is high, the binding of c‐di‐GMP to LapD promotes the interaction between LapD and LapG, thereby inhibiting LapA from being processed and facilitating surface adhesion or biofilm stabilization (Figure 1A)^6^, ^27^. When intracellular c‐di‐GMP levels are low, LapD does not bind to and sequester LapG, allowing LapG to process and release LapA from the cell surface, which promotes bacterial planktonic growth or biofilm dispersion (Figure 1B)^6^, ^27^. Therefore, LapD is regulated by global c‐di‐GMP signaling (Figure 1A,B)^27^, ^49^. PDE RapA (regulator of adherence by phosphate) decreases intracellular c‐di‐GMP concentrations in response to low phosphate signals; as a result, LapD is unable to bind to c‐di‐GMP, preventing LapD from sequestering LapG and leading to the cleavage of LapA from the outer member^49^. LapD also responds to local c‐di‐GMP signaling (Figure 1C)^27^, ^50^. GcbC (di‐guanylate cyclase promoting biofilm) is an inner‐membrane‐spanning DGC that responds to extracellular citrate by producing c‐di‐GMP, contributing to the local c‐di‐GMP pool^50^. Citrate stimulates GcbC to produce c‐di‐GMP, which is then physically delivered to LapD via contact between GcbC and LapD^27^, ^43^, ^50^, ^51^. In short, LapD regulates surface adhesion and biofilm maintenance in response to both global and local c‐di‐GMP signaling^27^. Adhesin LapA, an essential component of the biofilm extracellular matrix, is directly controlled by the c‐di‐GMP effector LapD. In this regulatory pattern, bacteria respond directly and efficiently to global or local c‐di‐GMP signaling to regulate biofilm formation. Other *Pseudomonas* species, such as *P. aeruginosa* ^52^, ^53^ and *P. putida* ^54^, as well as some other γ‐proteobacteria, such as *Legionella pneumophila* ^46^, ^55^, *V. cholerae* ^56^, ^57^, and some *Shewanella* species^58^, ^59^, have Lap systems with a regulatory model that is quite similar to that of *P. fluorescens* ^27^, suggesting that the Lap system is a crucial regulatory system for c‐di‐GMP to govern biofilm formation in some γ‐proteobacteria. In addition to these c‐di‐GMP effectors with degenerate GGDEF or EAL domains, several c‐di‐GMP metabolic enzymes with intact GGDEF or EAL domains also function as c‐di‐GMP effectors when the enzyme activity domain is not activated. RpfR (regulation of pathogenicity factors) is not only a PDE but also a c‐di‐GMP effector in *Burkholderia cenocepacia*. At high cell density, the concentration of the quorum sensing signal BDSF (*Burkholderia* diffusible signal factor) increases significantly, which binds to RpfR and triggers its c‐di‐GMP PDE activity. RpfR decreases intracellular c‐di‐GMP to extremely low levels, allowing for the formation of the BDSF–RpfR–GtrR (global transcriptional regulator downstream RpfR) complex. The ternary complex (BDSF–RpfR–GtrR) binds to the target promoter DNA and enhances the expression of virulence‐associated genes. At low cell density, the PDE activity of RfpR cannot be activated due to low concentrations of BDSF. When c‐di‐GMP level is significantly high, RpfR functions as a c‐di‐GMP effector by binding to c‐di‐GMP. Although the c‐di‐GMP‐RpfR can still bind to GtrR, the ternary complex (c‐di‐GMP–RpfR–GtrR) cannot bind to target promoter DNA^60^. Although there are few examples of c‐di‐GMP metabolic enzymes acting as c‐di‐GMP effectors, the RpfR regulatory model shows that as the signal to activate a c‐di‐GMP metabolic enzyme activity is lost, it can still function as a c‐di‐GMP effector to control physiological processes. Consequently, c‐di‐GMP metabolic enzymes are potentially targets in future studies looking for c‐di‐GMP effectors.

It was discovered that several proteins with various roles, such as histidine kinase, elongation factor, and deacetylase, are c‐di‐GMP effectors. The life cycle of *Caulobacter crescentus* is unique in that it asymmetrically divides into a sessile stalked (ST) cell that enters the S‐phase and a motile swarmer (SW) cell that stays in the G1 phase^61^ *. C. crescentus* separates its cell cycle stages strictly, which are regulated by cytoplasmic c‐di‐GMP levels^62^. Bacteria are halted in the G1 phase when intracellular c‐di‐GMP levels are very low, whereas cytoplasmic c‐di‐GMP is at an intermediate level during the rest of the cell cycle^63^, ^64^. Multiple c‐di‐GMP effectors are involved in the G1/S transition, including two histidine kinases, stalk biogenesis histidine kinase A (ShkA) and cell cycle kinase A (CckA)^65^, ^66^. In most conditions, a histidine kinase and its cognate response regulator, form a two‐component signal transduction system^67^. After being activated by a specific signal, histidine kinase undergoes autophosphorylation^67^, ^68^. The phosphoryl group is then transferred to the cognate response regulator, which could be a transcription factor or a functional protein that regulates gene expression or interacts with other proteins to influence physiological processes^67^, ^68^, ^69^. ShkA is a cytoplasmic hybrid histidine kinase, which is part of a three‐component signal transduction system that also includes the phosphotransferase protein ShpA (stalk biogenesis histidine phosphotransferase A) and the transcription factor TacA (transcriptional activator A)^70^, ^71^. ShkA harbors four domains: a dimerization histidine‐phosphotransfer domain (DHp), a catalytic domain (CA), a pseudo‐receiver domain (REC1), and a C‐terminal receiver domain (REC2)^65^. The CA domain binds ATP and transfers the phosphoryl group from ATP to the conserved His residue of DHp, completing the autophosphorylation process of ShkA^65^. ShkA has a unique active model. Generally, the N‐terminus of histidine kinases frequently contains a signal‐sensing domain that recognizes intracellular signals or external stimuli and promotes autophosphorylation^67^, ^68^. However, due to the absence of a signal‐sensing region at the N‐terminus, ShkA autophosphorylation cannot be initiated. A further restriction on ShkA autophosphorylation is provided by the linker region between REC1 and REC2^65^. As a result, when intracellular c‐di‐GMP levels are low, ShkA kinase activity is inhibited, thereby halting the bacteria in the G1 phase^65^. When DGC PleD (pleiotropic gene D) is activated, intracellular c‐di‐GMP levels rise significantly^72^. The restriction on autophosphorylation of ShkA is removed by binding to c‐di‐GMP, which means that the histidine kinase activity of ShkA is specifically activated by c‐di‐GMP^65^. Subsequently, the phosphoryl group is transferred to TacA by ShpA, which triggers TacA's transcriptional regulatory activity and induces the expression of G1/S‐specific genes^65^, ^71^, ^73^.

Two‐component regulatory systems are centered on the phosphorylation and dephosphorylation pathways^74^. In some circuits, the histidine kinase is only involved in cognate response regulator phosphorylation, while another phosphatase is in charge of response regulator dephosphorylation^74^. However, the histidine kinase often controls the phosphorylation and dephosphorylation of its cognate response regulator in most two‐component systems^74^, ^75^. By modifying one or both of these processes, the input stimulus for these bifunctional histidine kinases regulates the ratio of kinase to phosphatase activity, thereby determining the level of phosphorylation in the response regulator^74^. Although the phosphatase activity of most bifunctional histidine kinases mainly buffers against cross‐phosphorylation^74^, ^75^, some histidine kinases' bifunctional activities can respond to distinct inputs and perform different regulatory roles^63^. The master regulator CtrA (cell cycle transcriptional regulator), which regulates the cell cycle stages in *C. crescentus*, can be activated or inactivated by the bifunctional histidine kinase CckA^66^, ^76^. CckA is also a c‐di‐GMP effector. c‐di‐GMP directly binds to CckA and inhibits kinase activity while promoting phosphatase activity^62^. Elevated levels of intracellular c‐di‐GMP at the G1/S transition allow CckA to switch from kinase to phosphorylation mode, enabling replication initiation and cell cycle progression^62^. Although c‐di‐GMP effectors ShkA and CckA are both histidine kinases, their regulatory mechanisms vary. Binding to c‐di‐GMP activates ShkA kinase activity, whereas binding to c‐di‐GMP converts CckA from kinase to phosphatase^62^, ^65^. The identification of some histidine kinases as c‐di‐GMP effectors represents a novel regulatory framework in bacterial regulation. Bacteria have an abundance of histidine kinases, which allow them to respond to a variety of signals. It is advantageous to integrate signals to regulate physiological processes once a histidine kinase can detect both environmental cues and intracellular c‐di‐GMP signals.

Recently, translation elongation factor P (EF‐P) was identified as a c‐di‐GMP effector in *Acinetobacter baumannii* ^30^. In proteins, a sequence with two or more consecutive proline residues (polyproline) results in the formation of the poly‐l‐proline helix of type II (PPII), which is predominantly localized in the solvent‐exposed regions and mediates protein–protein and protein–nucleic acid interactions^77^. Thus, the PPII motif in pathogens is important in mediating host–pathogen interactions^78^. Because proline, unlike all other natural amino acids, has a pyrrolidine ring, the polyproline causes ribosome stalling during translation, which is alleviated by EF‐P in prokaryotes^79^, ^80^. In *A. baumannii*, although EF‐P has a basal level of activity in the absence of c‐di‐GMP, its function is greatly enhanced when c‐di‐GMP is present^30^. EF‐P promotes the translation of virulence or pathogenesis proteins containing consecutive proline residues by interacting with c‐di‐GMP, facilitating host–pathogen interactions, and leading to chronic host infection^30^. *A. baumannii* is one of the most serious and antibiotic‐resistant opportunistic nosocomial pathogens^81^, ^82^. Biofilm formation of *A. baumannii* is one of the determinants of virulence^82^. Similar to other Gram‐negative bacteria, *A. baumannii*'s “lifestyle transition” from motility to biofilm is regulated by c‐di‐GMP level^83^. When intracellular c‐di‐GMP levels are high, *A. baumannii* tends to form a biofilm to cause chronic host infection^83^. In addition, c‐di‐GMP increases host–pathogen interaction and further worsens host chronic infection by enhancing EF‐P function^30^. Thus, the simultaneous activation/inhibition of multiple c‐di‐GMP effectors with varied regulatory functions allows bacteria to coordinate and integrate various physiological processes in response to fluctuating environmental stimuli. EF‐P is found in a variety of bacterial species, and this type of EF‐P in *A. baumannii* is highly conserved in several species, such as *Acinetobacter, Halomonas, Pseudoalteromonas, Vibrio, Xanthomonas*, and *Yersinia* ^30^. The finding that EF‐P is a unique c‐di‐GMP effector may inspire more research into the functions and mechanisms of EF‐P in c‐di‐GMP signaling systems in diverse bacterial species.

**Figure 2:**
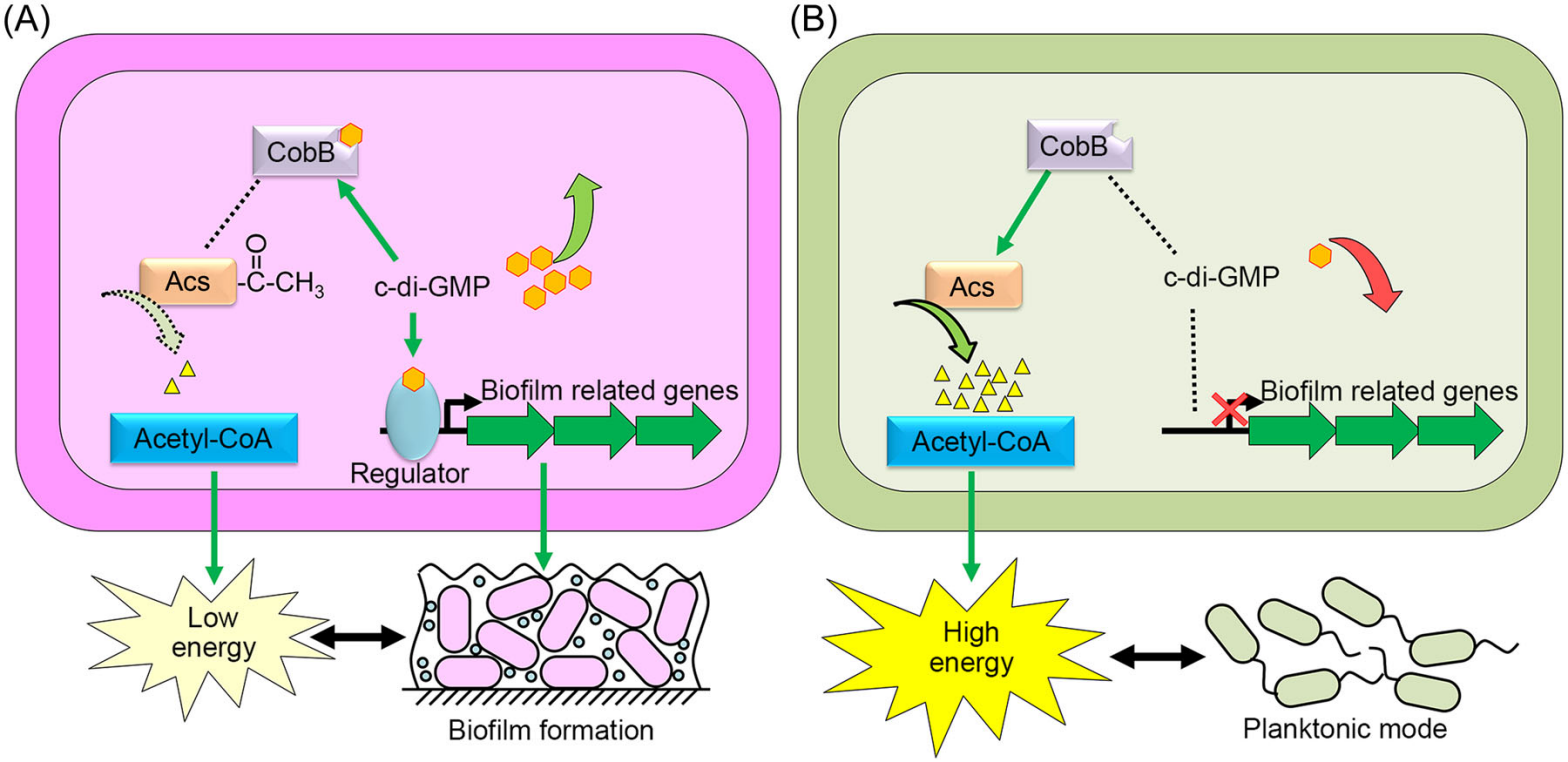
The regulatory model that c‐di‐GMP integrates cell energy and lifestyle in *Escherichia coli*. (A) High intracellular c‐di‐GMP levels are associated with biofilm formation. High intracellular c‐di‐GMP levels repress the deacetylase activity of CobB, leading to low‐energy metabolism. Thus, bacteria in biofilms exhibit slow cell growth and proliferation to support survival. (B) Low intracellular c‐di‐GMP levels tend to facilitate a planktonic lifestyle. Low c‐di‐GMP levels eliminate the inhibition of CobB's deacetylase activity, resulting in high‐energy metabolism. Thus, planktonic bacteria exhibit rapid cell growth and proliferation. Solid arrow indicates direct regulation; dashed line indicates nonregulation.

Another c‐di‐GMP effector example is the deacetylase CobB of *Escherichia coli*. CobB is an NAD^+^‐dependent deacetylase that is highly conserved in prokaryotes^84^, ^85^. It exists in two isoforms: CobB_L_, which has 273 amino acids, and CobB_S_ (236 amino acids), which lacks the 37‐amino‐acid N‐terminal tail of CobB_L_ ^86^. CobB_L_ is a c‐di‐GMP effector, and c‐di‐GMP inhibits its deacetylase activity by binding to the R[8]X8R[17]X3E[21] motif at the N‐terminus^87^. In *E. coli*, CobB_L_ is the sole known protein deacetylase, controlling the deacetylation of acetyl‐CoA synthetase (Acs) and many other important proteins^88^, ^89^, ^90^. Acs catalyze the synthesis of acetyl‐CoA, which is a central component of energy metabolism^87^, ^88^. Acs activity is regulated by acetylation. Specifically, acetylation inhibits Acs synthase activity, which is activated by CobB deacetylation^88^. c‐di‐GMP controls the synthesis of acetyl‐CoA by regulating the deacetylase activity of CobB, which links intracellular energy metabolism to bacterial lifestyle (Figure 2)^87^. When intracellular c‐di‐GMP levels significantly rise, bacteria form biofilms^6^. High intracellular c‐di‐GMP levels repress the deacetylase activity of CobB, which decreases acetyl‐CoA synthesis by inhibiting the deacetylation of Acs^87^. The low intracellular acetyl‐CoA concentration leads to low‐energy metabolism, slow cell growth, and proliferation in biofilm (Figure 2A). Low intracellular c‐di‐GMP levels inhibit biofilm and activate motility‐related genes, allowing bacteria to grow in a planktonic state^6^, ^9^. In addition, low c‐di‐GMP levels eliminate the inhibition of CobB's deacetylase activity, which deacetylates Acs to generate acetyl‐CoA^87^. A high intracellular acetyl‐CoA concentration indicates that cells have enough energy to support bacterial planktonic development (Figure 2B). It is well known that c‐di‐GMP directly regulates the genes and proteins involved in the synthesis of the biofilm matrix. However, the transition from planktonic growth to biofilm necessitates the coordination of all physiological functions in cells, rather than just the synthesis of extracellular matrix, which allows cells to adapt to their environment by switching their physiological metabolism from a rapid growth and proliferation status to a survival status.

The conservative domain enables the identification of all potential DGCs/PDEs in various bacteria, resulting in the regulatory functions of these DGCs/PDEs being well investigated in some bacteria^15^, ^23^, ^91^, ^92^, ^93^. Furthermore, the mechanisms of several c‐di‐GMP effectors containing conserved domains and some classic c‐di‐GMP effectors have been thoroughly investigated^48^, ^94^, ^95^. Numerous reviews have extensively detailed advances in both of the mentioned topics^6^, ^9^, ^14^, ^23^, ^27^, ^96^, ^97^. However, since most c‐di‐GMP effectors lack a conserved domain, our knowledge of the mechanical details of most c‐di‐GMP effectors is limited. Through multiple c‐di‐GMP effectors, c‐di‐GMP participates in regulating a variety of physiological processes, fulfilling its function as a global regulatory factor. Thus, finding c‐di‐GMP effectors is an extremely essential topic for research.

The examples above represent only a small portion of advances in c‐di‐GMP effector research, which does not eliminate the significance of the c‐di‐GMP effectors that are not mentioned. In contrast, a thorough understanding of each c‐di‐GMP effector's regulatory model is required to gain a better understanding of how c‐di‐GMP functions as a global regulatory factor. We provide these instances above to highlight two issues. First, by binding to various c‐di‐GMP effectors such as riboswitch, transcription factor, and regulatory protein, the c‐di‐GMP signal regulates biological processes at the transcriptional, posttranscriptional, translational, and posttranslational levels. Second, although the links between intracellular c‐di‐GMP levels and the transition of bacterial lifestyles have been well established in some bacteria, research on how c‐di‐GMP affects other physiological systems, such as central and secondary metabolism, is limited. The proteins with significant roles, such as anti‐σ factor, histidine kinase, elongation factor, and deacetylase, have been identified as c‐di‐GMP effectors in certain bacteria^26^, ^39^, ^65^, ^87^, which advance the regulation of c‐di‐GMP in central and secondary metabolism. Furthermore, since these proteins are widespread or conserved across many bacterial species, it is possible that they are also used by other species as c‐di‐GMP effectors. Thus, finding more c‐di‐GMP effectors and learning about their biological functions will allow us to better understand the global regulatory functions of c‐di‐GMP in future research, revealing how bacteria effectively coordinate various physiological processes via c‐di‐GMP.

## THE GLOBAL REGULATION OF cAMP IN BACTERIA

cAMP is a universal second messenger found in all organisms. In bacteria, adenylate cyclase catalyzes the formation of cAMP from ATP, whereas cAMP PDE catalyzes its breakdown^98^, ^99^. Most bacteria have significantly fewer cAMP metabolic enzymes than c‐di‐GMP metabolic enzymes. For instance, one to three adenylate cyclases and one cAMP PDE are present in γ‐proteobacteria^98^, ^99^, ^100^. cAMP exerts its global regulatory function via CRP in most bacteria^98^, ^100^. In *E. coli*, cAMP–CRP was discovered to be involved in carbon catabolite repression (CCR)^101^. When a preferred carbon source is available, it is transported into the cell and concomitantly phosphorylated by the phosphoenolpyruvate: carbohydrate phosphotransferase system (PTS). The phosphorylation status of the PTS is lower in the presence of the preferred carbon source since the phosphoryl group is transferred to a preferred carbon source to complete its transport into the cell^101^, ^102^. In the absence of a preferred carbon source, phosphorylated PTS proteins accumulate and transfer a phosphoryl group to adenylate cyclase, which is activated to produce cAMP^101^, ^102^. cAMP binds to and activates CRP, which triggers the transcription of genes encoding non‐PTS transport enzymes to transport and hydrolyze these non‐PTS substrates^101^, ^102^. In addition to participating in CCR, cAMP–CRP serves as a global transcription factor, controlling the transcription of numerous genes^103^. Its regulatory model is well‐established in *E. coli*: apo‐CRP (homodimer CRP in the absence of cAMP) binds to DNA in a nonspecific and weak manner; CRP binds DNA as specifically and strongly as it forms a complex with cAMP^104^. cAMP–CRP interacts directly with RNA polymerase to promote transcription initiation^105^.

Despite its well‐known role in sensing the availability of carbon and controlling nutrient consumption by bacteria, cAMP–CRP is an essential regulator of virulence in many pathogens^101^, ^106^, ^107^, ^108^, ^109^. In *Yersinia enterocolitica*, for example, cAMP–CRP is necessary to control not only CCR‐related genes but also virulence genes encoding plasminogen activator and type III secretion system (T3SS)^110^. Despite high homology with CRP_*E. coli*_, CRP occurs in *Pseudomonas* through mechanisms that are entirely alien to CRP*_E. coli_* ^109^, ^111^. Instead of cAMP–CRP, catabolite repression control (Crc) proteins regulate CCR in *P. aeruginosa*, and the homolog of CRP is known as virulence factor regulator (Vfr) due to its specific role in the control of virulence genes^109^, ^112^. Vfr is 67% identical and 91% similar to the CRP of *E. coli* ^109^. Although Vfr can influence gene transcription in a cAMP‐independent manner, cAMP is usually required to activate Vfr's transcriptional regulatory action^113^. cAMP–Vfr mediates biofilm formation and various virulence systems in response to solid surfaces^114^. When planktonic bacteria come into contact with a solid surface, the flagellar load of the bacterial flagellum increases significantly, resulting in an interaction between FlhF (flagellar biosynthesis protein F) and FimV (fimbriae gene V)^114^. The interaction raises the intracellular cAMP level by activating both adenylate cyclases CyaA and CyaB^113^. cAMP stimulates the transcriptional regulatory activity of Vfr, and cAMP–Vfr induces the expression of several genes involved in irreversible attachment, thus contributing to biofilm development^109^, ^113^, ^114^. Furthermore, although *P. putida* CRP is 62% identical and 80% similar to its *E. coli* equivalent, cAMP–CRP*_P. putida_* regulates the utilization of various amino acids and dipeptides as nitrogen sources rather than CCR^115^, ^116^. In *P. putida*, a phenylalanine hydroxylase involved in the conversion of l‐phenylalanine into l‐tyrosine is encoded by the *phhAB* operon^117^. The specific phenylalanine hydroxylase regulator (PhhR) functions as an enhancer protein to promote *phhAB* operon transcription^117^, ^118^. As the binding sites between PhhR and RNA polymerase in the *phhAB* promoter are too far apart, a cAMP–CRP complex is needed to activate transcription^111^, ^117^, ^119^. Simultaneous binding of cAMP–CRP and PhhR bends DNA, bringing RNA polymerase and positive regulators into close proximity^111^. This improves the transcription of *phhAB*, resulting in the production of l‐tyrosine^111^. Remarkably, both *vfr* and *crp_P. putida_* fully complement the *crp_E. coli_* deletion mutant, but neither the *vfr* nor the *crp_P. putida_* deletion mutant can be complemented by *crp_E. coli_* ^109^, ^115^, ^120^. Why? It is possible that different bacteria have distinct intracellular CRP protein concentrations and cAMP levels, resulting in diverse CRP and cAMP binding mechanisms. CRP in *P. putida* cells is significantly less abundant than that in *E. coli*.^115^ Furthermore, poor adenylate cyclase mRNA translation and a strong functional cAMP–PDE lead to a low cAMP level in *P. putida*, which necessitates an ultratight affinity for CRP*_P. putida_* to bind cAMP^120^, ^121^. Therefore, the cAMP level in *E. coli* cells is significantly higher than that in *P. putida*, which is sufficient to bind to the *P. putida* CRP homolog^120^, ^121^. In *P. putida*, however, the intracellular cAMP level is insufficient to support the binding of CRP*_E. coli_* ^115^, ^120^, ^121^. Due to the low cAMP level in *P. putida* cells, CRP*_P. putida_* binds one cAMP molecule per protein dimer, as different from *E. coli*, where the binding stoichiometry between cAMP and CRP is 1‐to‐1^120^, ^121^, ^122^, ^123^.

CRP is one of the cAMP effectors in *Mycobacterium tuberculosis*, which is 32% identical to the CRP of *E. coli* ^122^. The CRP regulatory model of *M. tuberculosis* can help us understand why the maintenance of a given regulatory device does not mean keeping the biological function(s) regulated by it^122^. In contrast to most γ‐proteobacteria, *M. tuberculosis* has 16 adenylate cyclase‐like proteins, 10 of which can increase intracellular cAMP levels in response to internal and/or external signals, and only one cAMP–PDE has inadequate PDE activity to degrade cAMP, resulting in a high intracellular cAMP level in this bacterium^124^, ^125^. Unlike *E. coli*, where cAMP enhances the binding affinity of CRP with sequence‐specific promoters, *M. tuberculosis* CRP (CRP_MTB_) affinities with DNA are identical in the presence and absence of cAMP due to the comparatively weak binding of CRP_MTB_ and cAMP^122^, ^126^. Thus, unlike previously published models for other CRPs, cAMP‐mediated transcriptional control is unique to *M. tuberculosis*.

The apo‐CRP_MTB_ dimer binds to both specific and nonspecific DNA sequences to generate high‐order CRP_MTB_‐DNA complexes in the absence of cAMP^127^. After macrophage infection, intracellular cAMP levels increase significantly, resulting in all apo‐CRP_MTB_ dimers being bound by a single cAMP molecule^127^. One cAMP is sufficient for the dissociation of high‐order CRP_MTB_‐DNA oligomers and disruption of nonspecific interactions, preserving only the specific DNA sequences bound^127^. When intracellular cAMP levels rise dramatically, each CRP_MTB_ dimer is saturated by two cAMP molecules, causing the transcription of a particular DNA promoter^127^. The importance of this regulatory paradigm for *M. tuberculosis* cannot be overstated. After infecting macrophages, *M. tuberculosis* generates plenty of intracellular cAMP, which is first secreted into macrophages, causing them to become intoxicated^122^. Thus, cAMP first serves as a virulence factor, which cannot trigger gene transcription until its intracellular concentration is extremely high^122^. Several strategies have been developed to prevent bacterial genes from being activated by cAMP too early, including low CRP_MTB_ concentrations in cells, the formation of high‐order CRP_MTB_‐DNA oligomers, and weak CRP_MTB_‐cAMP binding^122^, ^127^.

The above illustrations demonstrate that variations in intracellular cAMP levels, CRP concentrations, and the affinities between cAMP and different CRP determine the diverse physiological roles of cAMP–CRP in different bacteria. However, since CRP is highly conserved, its global regulation models are similar in most bacteria. cAMP–CRP controls the transcription of numerous genes as a global transcription factor to control a variety of biological processes.

## COMPARISON OF c‐di‐GMP AND cAMP REGULATORY MODELS

Bacteria sense environmental stimuli via membrane‐bound receptors that transduce signals internally^17^. Some information flow typically converges on nucleotide second messengers^17^. Both cAMP and c‐di‐GMP serve as global regulators in bacteria, and their regulatory mechanisms have several parallels. The synthetase/PDE controls levels of both cAMP and c‐di‐GMP in response to internal and environmental cues^9^, ^15^, ^101^, ^102^. Additionally, both cAMP and c‐di‐GMP typically form complexes with their effectors that control the signal transduction pathways downstream^9^. However, the global regulatory frameworks of cAMP and c‐di‐GMP are considerably different in most bacteria. This discrepancy is mostly due to the fact that there are more c‐di‐GMP metabolic enzymes and effectors than cAMP in most bacteria. Numerous adenylate cyclases and cAMP effectors are present in some bacteria, however, including *M. tuberculosis* ^124^, ^128^, ^129^, ^130^. Therefore, it is essential to carefully evaluate the differences in global regulation between c‐di‐GMP and cAMP as well as the reasons for those differences.

**Figure 3:**
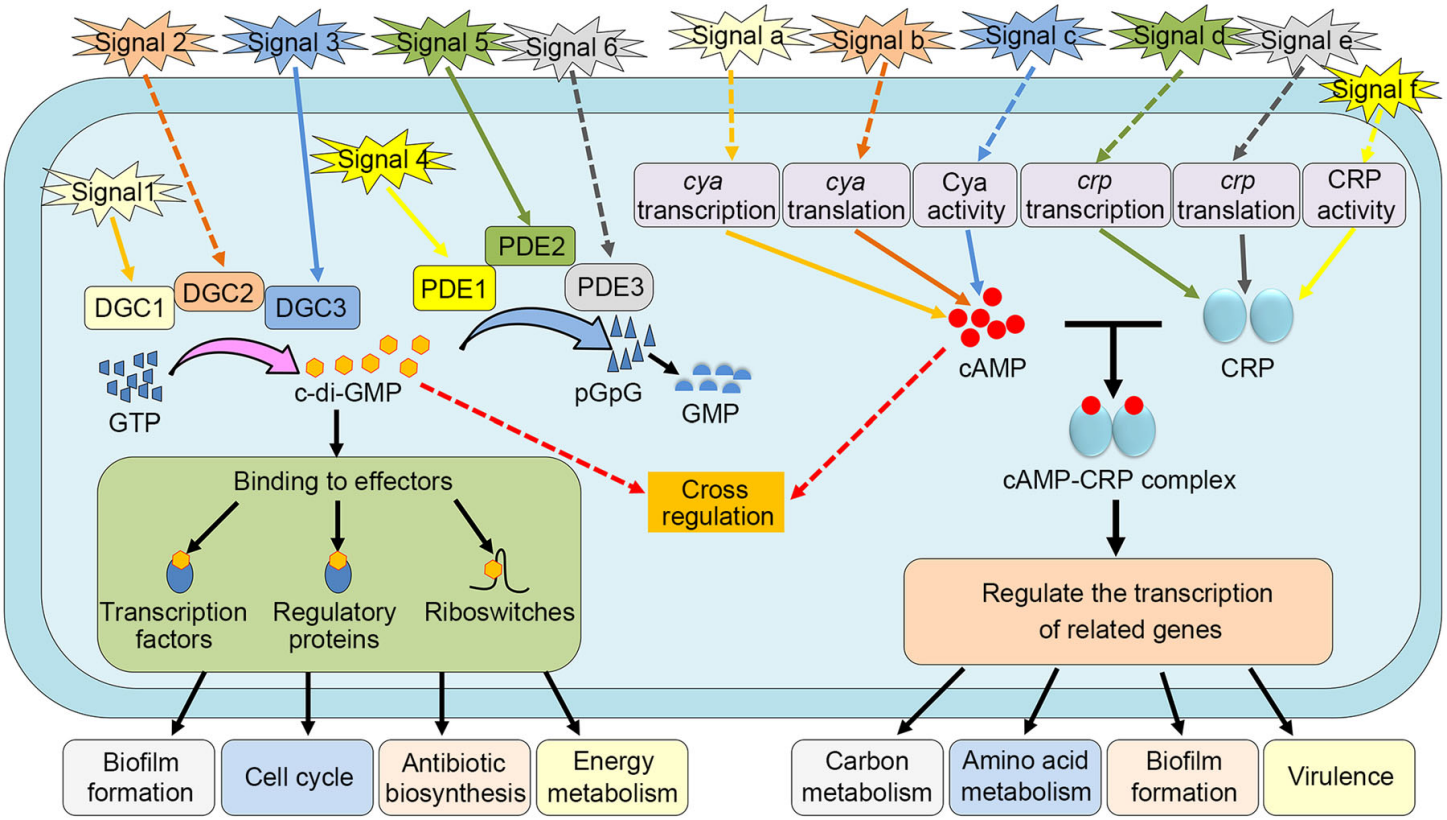
The different regulatory frameworks between c‐di‐GMP and cyclic AMP (cAMP). Different signals control intracellular c‐di‐GMP levels via a large number of c‐di‐GMP metabolic enzymes/PDEs (DGCs/PDEs), and c‐di‐GMP exerts its global regulation through a huge number of effectors with varying activities. cAMP metabolic enzymes are controlled at multiple levels, allowing intracellular cAMP levels to respond to more signals. The major cAMP effector, CRP, is controlled at various levels and exerts its global regulation primarily via controlling the transcription of numerous genes. Signals 1‐6 and a–f represent different signals. DGCs and PDEs can be located in the cytoplasm or on the inner membrane. Solid arrow indicates direct regulation. Dashed arrow indicates indirect regulation. CRP, cAMP receptor protein; DGCs, di guanylate cyclases; PDEs, phosphodiesterase.

We evaluate the differences in how metabolic enzymes of both nucleotide second messengers respond to intracellular/extracellular signaling (Figure 3). Most bacteria contain a large number of c‐di‐GMP metabolic enzymes^15^, ^23^, ^91^, ^92^. The activity of most DGCs/PDEs depends not only on GGDEF or EAL/HD‐GYP domains but also on signal input via an N‐terminal sensory domain^15^, ^131^, ^132^. Internal and external cues stimulate the N‐terminal sensory domain of DGCs and PDEs to initiate the enzyme activities that control intracellular c‐di‐GMP levels^9^, ^131^, ^132^, ^133^, ^134^. In conclusion, different signals control intracellular c‐di‐GMP levels via different DGCs/PDEs. Given the abundance of c‐di‐GMP metabolic enzymes, a variety of signals can be responded to^9^. However, cAMP is distinct from c‐di‐GMP. Since most bacteria, especially γ‐proteobacteria, only contain a small number of cAMP metabolic enzymes, the internal/external signals that directly activate these enzymes are limited. Thus, cAMP metabolic enzymes are controlled at multiple levels, allowing intracellular cAMP levels to respond to more signals^101^, ^102^, ^114^, ^121^, ^135^, ^136^, ^137^, ^138^. First, cAMP metabolic enzymes can be regulated at the transcriptional level. For instance, a temperature signal of 37°C regulates the transcription of *cyaA* by activating the two‐component system BvgAS (*Bordetella* virulence gene) in *Bordetella pertussis* ^136^, ^137^, ^138^. In addition, cAMP metabolic enzymes can be controlled at the translational level. In *P. putida*, a lack of a high‐quality Shine–Dalgarno (SD) sequence may hinder the establishment of the translation initiation complex properly, impeding effective translation of the *cyaA* mRNA^121^. Moreover, the activity of adenylate cyclases can be directly regulated by some signals. For instance, CyaA in *E. coli* is activated by the high phosphorylation level of PTS system brought on by low glucose concentration, while solid surface signals boost the enzyme activities of CyaA and CyaB in *P. aeruginosa* ^101^, ^102^, ^114^. Certainly, several c‐di‐GMP metabolic enzymes are also regulated at different levels. In *Shewanella putrefaciens* CN32, PDE LrbR (lactate‐dependent repressor for biofilm formation regulator) decreases intracellular c‐di‐GMP levels to negatively regulate biofilm formation in response to carbon source signals^131^. LrbR is part of a three‐component signal transduction system that also includes the histidine kinase LrbS and the transcription factor LrbA^131^. LrbS activates the cognate regulators LrbR and LrbA by transferring the phosphoryl group to them^131^. The activated LrbA promotes the transcription of *lrbR* ^131^. In short, LrbR is regulated at the transcriptional and posttranslational levels by LrbA and LrbS to allow it to respond more accurately to carbon source signals rather than to a variety of signals. c‐di‐GMP depends on a great number of metabolic enzymes to respond to numerous signals, whereas distinct signals regulate cAMP metabolic enzymes at different levels. Therefore, even though the number of cAMP metabolic enzymes is much lower than that of c‐di‐GMP metabolic enzymes, intracellular cAMP levels can still be controlled by a variety of signals.

Although both cAMP and c‐di‐GMP typically interact with effectors to exert regulatory effects, their regulatory frameworks differ significantly in most bacteria (Figure 3). Bacteria have various c‐di‐GMP effectors with diverse functions, which respond to both global and local c‐di‐GMP signaling^9^, ^17^, ^87^. In global c‐di‐GMP signaling, since the affinities (*K*_d_) of different c‐di‐GMP‐binding effector components vary, they are progressively activated as intracellular c‐di‐GMP levels gradually increase, controlling the pertinent signal transduction pathway^9^, ^49^. The local c‐di‐GMP production is physically delivered to the effector via a direct interaction between the local DGC/PDE and the c‐di‐GMP effector in local c‐di‐GMP signaling^9^, ^17^, ^27^, ^50^. The presence of numerous c‐di‐GMP effectors with a variety of roles not only allows c‐di‐GMP to regulate the same biological process at different levels, such as transcriptional and translational regulation or allosteric regulation, but also allows c‐di‐GMP to carry out its global regulatory role on a wide range of biological functions^6^, ^9^. Compared to c‐di‐GMP, cAMP has a completely distinct regulatory mechanism. Since cAMP's global regulatory function is primarily performed by CRP, controlling CRP is essential for governing the global regulation of cAMP. Thus, in most bacteria, CRP is regulated at transcriptional, posttranscriptional, and posttranslational levels^107^, ^139^, ^140^. First, the amount of CRP in the cell is crucial for the complex's regulatory function. For instance, *P. putida* cells have significantly lower CRP concentrations than *E. coli* cells^115^. In *Yersinia pestis*, the PhoPQ (alkaline phosphatase gene P‐Q) two‐component system directly regulates *crp* transcription, while the small RNA chaperone Hfq (HF‐I for phage Qβ) regulates CRP synthesis at the posttranscriptional level^107^, ^139^. Second, the carbon source signal can control the acetylation of the lysine (K100) in CRP, thus regulating the transcriptional regulation activity of CRP^141^. Third, although cAMP–CRP is a global regulator that controls the transcription of various genes, this regulation does not take place simultaneously or in a uniform manner. For example, the position (relative to the transcription start site) of cAMP–CRP binding to various promoters is quite flexible, resulting in a diversity of cAMP–CRP and RNA polymerase interaction models that influence RNA polymerase's ability to initiate transcription^105^. Finally, the transcriptional regulatory activity of cAMP–CRP can be influenced by specific transcription factors. At CytR‐repressed promoters, the transcription factor CytR represses transcription by directly interacting with CRP (the activator) to inhibit CRP‐dependent activation^142^. Essentially, CRP is a common transcription factor whose regulatory frameworks parallel those of some c‐di‐GMP effector transcription factors. For instance, the transcriptional regulatory activity of FleQ is controlled not only by its second messenger ligand but also by the antiactivator FleN in *P. aeruginosa* ^8^, ^29^, ^143^. Furthermore, similar to CRP, FleQ functions as a master transcription factor that regulates the transcription of numerous genes^8^, ^29^. However, c‐di‐GMP operates as a global regulatory factor not only via transcription factor effectors but also via a variety of other functional effectors. For example, in addition to FleQ, c‐di‐GMP regulates physiological processes in *P. aeruginosa* through multiple nontranscription factor effectors such as FlgZ (flagellar gene Z), BdlA (biofilm dispersion locus), and FimX (fimbriae gene X)^23^, ^144^, ^145^, ^146^, ^147^. In contrast to c‐di‐GMP, cAMP can only exert global regulatory function via CRP in most bacteria^98^. Despite the presence of another cAMP effector, cAMP‐binding protein CbpA, Vfr remains the primary effector for cAMP to exert its global regulatory function in *P. aeruginosa* ^100^, ^148^.

Recent research has shown that by relocating in the cell, CRP can influence the transcription activity of a certain transcription factor, hence controlling the transcription of genes regulated by that transcription factor^26^. A DNA‐binding aminopeptidase, PepA (peptidase A), serves as a global transcription factor to control the expression of 700 genes related to biofilm formation and motility/chemotaxis in *V. cholerae* ^140^, ^149^. When bacteria grow in nutrient‐replete conditions, CRP is directly associated with the cell membrane^140^. PepA is prevented from binding to DNA by localizing on the cell membrane via an interaction with CRP^140^. Under nutrient‐poor conditions, CRP and PepA are released from the membrane, which allows PepA to control related‐gene transcription, thereby aiding the bacteria to adapt to nutrient‐stress environments^140^. This work uncovers the regulatory functions of CRP at the posttranslational level. Although it has been demonstrated that a number of transcription factors localize on the cell membrane, their regulatory paradigms are diverse^150^, ^151^, ^152^, ^153^. First, sequestration to the membrane inhibits the transcription factor's transcriptional regulatory activity, which is then activated upon dissociation from the membrane^150^. For instance, when the transcription factor PutA (utilization of proline A) is present in the cytoplasm of *Salmonella typhimurium*, PutA represses the transcription of its target genes; however, PutA loses its transcriptional regulatory function when it associates with the membrane^150^. Second, the transcription factor is a membrane‐integrated protein that remains on the cell membrane^151^. The signal to which it responds, rather than its localization, activates its transcriptional regulatory function^151^. Cadaverine biosynthesis C (CadC) is an example of a membrane‐integrated transcription factor from *E. coli* that responds to low environmental pH by activating pH tolerance genes^151^. Third, the transcription factor interacts with other membrane‐integrated proteins to enable indirect localization to the membrane, where the membrane‐integrated proteins regulate the regulatory activity of transcription factors^152^. The c‐di‐GMP effector Clp (CRP‐like protein) is a transcription factor in *Lysobacter enzymogenes*, and c‐di‐GMP mediates its transcriptional regulatory function^152^. Clp interacts with LchP, an inner‐membrane‐spanning PDE^152^. LchP is a weak c‐di‐GMP PDE, and Clp binding stimulates LchP's PDE activity, after which LchP degrades c‐di‐GMP from the Clp‐c‐di‐GMP complex to regulate Clp's transcriptional regulatory activity^152^. However, CRP is distinct from all three of the preceding situations. Membrane association does not interfere with CRP–DNA association or CRP's ability to control transcription, but it has a significant impact on PepA's ability to control transcription^140^. In addition to *V. cholerae*, cAMP–CRP can play regulatory roles at the posttranslational level in *S. putrefaciens* CN32^59^. Despite acting as a posttranslational regulator, cAMP–CRP achieves its global regulatory function primarily through its transcriptional regulatory action. In summary, these studies demonstrate that while both serve as global regulatory factors, cAMP and c‐di‐GMP have quite different regulation frameworks. c‐di‐GMP regulates various signal transduction pathways through its huge number of effectors with varying activities. In contrast, the major cAMP effector, CRP, is controlled at various levels and mediates a variety of physiological functions, primarily by controlling the transcription of numerous genes (Figure 3).

We hypothesize that the different regulation models of cAMP and c‐di‐GMP are due to the fact that both evolved in different ways in bacteria. cAMP–CRP may have initially controlled only a single or a few biological functions in the ancestors of some bacteria^115^. When bacteria enter a new habitat or their living environment changes, they must adapt to new environmental cues. It goes without saying that using the original regulators rather than creating new ones is the quickest way to mediate these physiological processes to assist bacteria in adapting to the new environment.^115^ As a result of controlling more physiological processes, cAMP–CRP evolves into a global regulator. The case with c‐di‐GMP is completely different. Initially, c‐di‐GMP may also control a few biological functions. To help bacteria adapt to new environments, certain physiological processes had to be incorporated into the c‐di‐GMP‐regulating signal transduction pathways. As a result, the essential protein for these physiological processes evolved into the c‐di‐GMP effector, and c‐di‐GMP became a global regulator.

## CROSS‐REGULATION BETWEEN cAMP AND c‐di‐GMP

**Figure 4:**
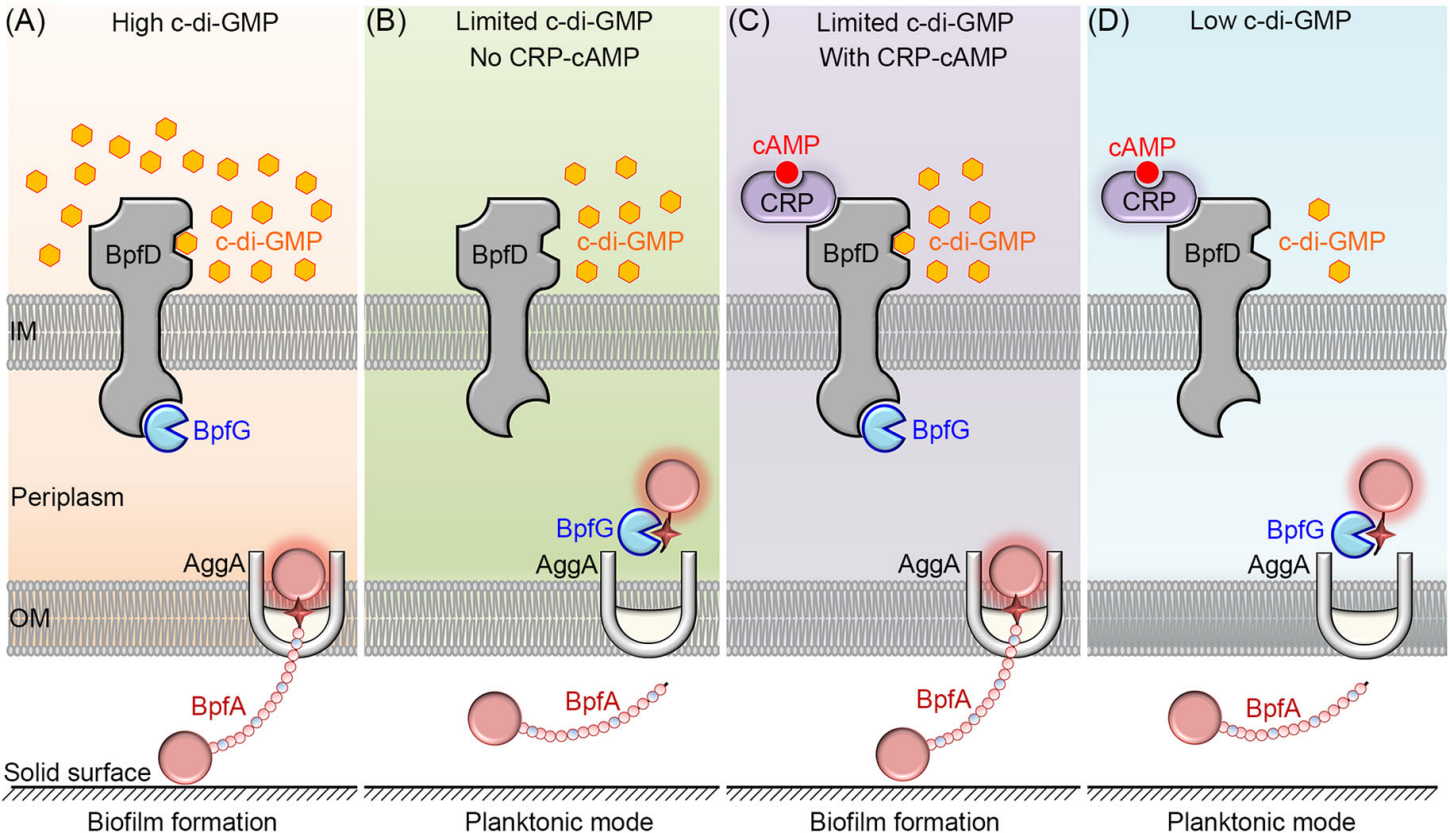
The cross‐regulation between cAMP and c‐di‐GMP in *Shewanella putrefaciens*. (A) High intracellular c‐di‐GMP levels support biofilm maintenance via the BpfAGD system. (B) Limited intracellular c‐di‐GMP levels lead to biofilm dispersion. (C) As intracellular c‐di‐GMP levels are limited, cAMP and c‐di‐GMP synergistically support biofilm maintenance through the direct interaction of their effectors, CRP and BpfD. (D) Low intracellular c‐di‐GMP levels below a certain threshold concentration lead to biofilm disperses.

Although extremely limited, the cross‐regulation of cAMP and c‐di‐GMP has recently advanced. For instance, in *P. putida*, c‐di‐GMP reduces intracellular cAMP levels since FleQ, a c‐di‐GMP effector, directly suppresses *cyaA* transcription^154^; cAMP–CRP decreases intracellular c‐di‐GMP levels by binding to the promoter of the DGC gene *gcsA* to prevent its transcription^155^. In addition, cAMP–Vfr inhibits the expression of the c‐di‐GMP effector gene *fleQ* in *P. aeruginosa* ^156^, and cAMP–CRP negatively regulates DGC, CdgA (cyclic‐di‐guanylate), and the c‐di‐GMP effectors VpsT and VpsR in *V. cholerae* ^157^. To summarize, cross‐regulation of c‐di‐GMP and cAMP is usually through control of the transcription of the effectors or metabolism enzyme genes of the other. According to a recent study, the cross‐regulation paradigm between cAMP and c‐di‐GMP is far more complex than previously thought. In *S. putrefaciens* CN32, the BpfAGD (biofilm‐promoting factor A‐G‐D) system regulates biofilm formation/maintenance in response to intracellular c‐di‐GMP levels (Figure 4)^59^. The regulatory model of the BpfAGD system resembles the *P. fluorescens* Pf0‐1 Lap system. When intracellular c‐di‐GMP levels are high, the interaction between c‐di‐GMP effector BpfD and periplasmic protease BpfG promotes BpfA localization to the cell surface, thereby supporting biofilm maintenance (Figure 4A)^59^. When intracellular levels of c‐di‐GMP are limited, the separation between BpfD and BpfG leads to BpfA being processed from the cell surface, resulting in biofilm dispersion (Figure 4B)^59^. In the presence of limited c‐di‐GMP, the binding of cAMP–CRP to BpfD strengthens the interaction between BpfD and BpfG, which maintains BpfA on the cell surface and thus supports biofilm maintenance (Figure 4C)^59^. Whether CRP and BpfD interact or not, the BpfD‐BpfG interaction cannot be maintained when intracellular c‐di‐GMP levels are low and below a certain threshold. As a result, BpfA is released from the cell surface, which leads to biofilm dispersion (Figure 4D)^59^. Many previous studies have shown that the regulatory effects of cAMP–CRP on biofilm formation are largely ancillary compared to c‐di‐GMP^158^. According to this regulation model, biofilm maintenance is supported by both cAMP and c‐di‐GMP^59^. In other words, the cAMP and c‐di‐GMP pathways cross‐regulate through direct interaction of their effectors, CRP, and an inner membrane‐spanning c‐di‐GMP effector BpfD to support biofilm maintenance synergistically^59^. This study presents an additional instance of how cAMP–CRP can function as a posttranslational regulator.

Cross‐regulation of cAMP and c‐di‐GMP is crucial for bacterial physiological processes. For instance, a key step in biofilm formation is for the bacterium to sense and respond to surfaces after initial attachment, which greatly enhances intracellular c‐di‐GMP levels^159^. However, how surface signals raise intracellular c‐di‐GMP levels is a critical issue in biofilm formation. In *P. aeruginosa*, swimming bacteria use their flagella to contact a solid surface, increasing the flagellar load^113^. This activates CyaA and CyaB, raising intracellular cAMP levels^113^. cAMP then activates its transcription factor effector Vfr, and cAMP–Vfr improves the transcription of Type IV pili (TFP) genes, including *pilY1* ^160^, ^161^. Cell surface‐associated PilY1 (pili structure gene Y1) influences the interaction between diguanylate cyclase SadC and the TFP alignment protein PilO to activate SadC (surface attachment‐defective), which increases intracellular c‐di‐GMP levels to promote irreversible initial attachment and bacteria entering the biofilm lifestyle^162^. In short, the flagellar system increases intracellular cAMP levels in response to the solid surface signal, and cAMP then raises the intracellular c‐di‐GMP levels via the pili system. Although cAMP increases intracellular c‐di‐GMP in the preceding example, the cross‐regulation between cAMP and c‐di‐GMP is actually quite complex. c‐di‐GMP has also been found to inhibit the cAMP‐Vfr signaling pathway in *P. aeruginosa*. As a result, additional experiments are required to reveal the regulatory models of these two nucleotide second messengers^163^. In summary, the cross‐regulation of cAMP and c‐di‐GMP plays an essential role in *P. aeruginosa*'s lifestyle transition from planktonic growth to biofilm formation. Many previous studies have shown that the regulatory effects of cAMP–CRP on biofilm formation are largely ancillary compared to c‐di‐GMP in most bacteria^158^. However, recent studies have demonstrated that cAMP and c‐di‐GMP play an equally important role in biofilm formation in some bacteria. In addition, although cAMP–CRP has been studied as a regulatory factor in bacteria for 60 years, its control of bacterial physiological metabolism may be more intricate than we realize.

Despite significant advances in our knowledge of the regulatory functions of cAMP and c‐di‐GMP, relatively little is currently known about the cross‐regulation between these two nucleotide second messengers. As the biological processes controlled by cAMP and c‐di‐GMP differ and sometimes overlap, research on cross‐regulation of cAMP and c‐di‐GMP can help to reveal how bacteria effectively coordinate and integrate different physiological processes via nucleotide second messengers.

## PERSPECTIVES

Nucleotide second messengers have been shown to be essential in regulating a variety of bacterial biological functions. Determining how they influence physiological processes is just one goal of research into their regulation. It is more important to explore how bacteria coordinate and integrate various physiological processes in response to intracellular and environmental signals. For instance, it is widely accepted that bacteria in biofilms exhibit slow cell growth and proliferation to support survival^164^. The identification that CobB is a c‐di‐GMP effector uncovers the underlying mechanism by which bacteria coordinate the biofilm formation with the maintenance of intracellular low‐energy metabolism. Thus, future research should focus on the following topics: (i) keep looking for the c‐di‐GMP effectors to learn more about regulatory models for c‐di‐GMP controlling bacterial physiological processes; (ii) look into the regulatory role and mechanism of cAMP–CRP as a posttranslational regulator in addition to the transcription factor; (iii) explore the cross‐regulation between cAMP and c‐di‐GMP or other nucleotide second messenger signaling pathways and the physiological significance of these cross‐regulation; and (iv) investigate the mechanisms by which nucleotide second messengers coordinates and integrates multiple physiological processes. In conclusion, nucleotide second messenger signaling remains one of the most active areas of molecular microbiology, with implications ranging from microbial ecology to human health.
